# The Effects of Sintering Temperature and Addition of TiH_2_ on the Sintering Process of Cu

**DOI:** 10.3390/ma12162594

**Published:** 2019-08-15

**Authors:** Huali Hao, Yanjing Wang, Hamid Reza Jafari Nodooshan, Yongyun Zhang, Shulong Ye, Yonghu Lv, Peng Yu

**Affiliations:** Department of Materials Science and Engineering, Southern University of Science and Technology, Shenzhen 518055, China

**Keywords:** copper, TiH_2_, sintering behavior, oxygen content, powder metallurgy

## Abstract

In this study, effects of sintering temperature and TiH_2_ on the sintering process of Cu are investigated. During sintering, the oxide in Cu decomposes and generates oxygen, which can become trapped in the material forming closed pores. Therefore, this results in low sintered density. Sintering behavior of Cu can be significantly improved by adding 0.5 wt.% TiH_2_ which decomposes during sintering, producing hydrogen and effectively reducing the oxide in Cu. Although gas products of the reduction reaction may still be trapped inside the close pores formed near the TiH_2_ particles and handicap the sintering of Cu, an isothermal treatment at 650 °C can avoid forming close pores. This allows reaction products to dissipate freely from the sample, subsequently increasing its sintered density.

## 1. Introduction

Copper is an important engineering material used in electrical and electronic industries, due to its excellent electrical and thermal conductivity [1,2,3,4,5,6,7,8]. Copper based composite materials, which contain hard ceramic particles aiming to either improve the tribological properties or decrease the coefficient of thermal expansion of copper matrix, have recently attracted the interest of many researchers [4,5,6,7,8]. These materials are difficult to machine and are mostly fabricated via powder metallurgy, which can provide net-shape fabrication [9,10,11,12]. 

Metal powders easily suffer from oxidation during manufacturing. Therefore, their oxygen contents are always higher than the base metal used to fabricate them. Oxygen is an interstitial element which has important influences on the sintering processes of metals and alloys. Oxygen has extremely low solubility in most structural metals such as Fe, Al and Cu [13,14]. Therefore, oxygen normally exists as oxides on the surface of those metal powders, handicapping their sintering process. Previous research on the sintering of Al alloys shows that the oxide on Al particles can prevent Al from sintering, and Al can only get sintered after more oxygen-affinitive elements such as Mg is introduced [15,16]. Mg can effectively reduce the aluminum oxide and remove it from the surface of Al particles at elevated temperatures [17,18,19]. Sintering of Cu is also influenced by the oxygen content in Cu powder [20,21,22]. 

Copper oxide can be reduced by hydrogen, consequently it is beneficial to sinter Cu in flowing hydrogen [1,23]. Our previous research indicates that when Cu is doped with 0.5% of elements, such as Al, Ti and Cr, oxides in the Cu can become reduced by them during sintering, subsequently improving its sinterability [24,25]. TiH_2_ is an intermediate product of the Ti fabrication industry. It decomposes and releases hydrogen when heated up. Furthermore, it is well known that TiH_2_ has surface cleaning effects during the dehydrogenation process; hydrogen atoms in TiH_2_ powder can remove the oxygen from Ti_2_O_3_ and TiO_2_ compounds [26,27]. Therefore, it is presumed that TiH_2_ can act as an oxygen scavenger to improve the sintering behavior of Cu. This may solve the problem of low sintering density from which many copper alloys and composites produced by powder metallurgy are suffering. In the present research, the effects of sintering temperature and TiH_2_ on the sintering process of Cu are investigated. The mechanisms of their influence are also discussed. This research provides a useful guide for selection of suitable sintering agents for Cu alloys.

## 2. Materials and Methodology 

Elemental powders of Cu (D_50_ = 10 μm, Sandvik Osprey Ltd., Neath, UK) and TiH_2_ (D_50_ = 45 μm, Wuyi Co. Ltd., Jiangsu, China) were used as raw materials. The Cu powders were blended with 0.5 wt.% TiH_2_ powders in a tubular mixer (Sinomix Sci. & Tech. Co., Sichuan, China) for 2 h (ethanol (Yonghua chemical Co. Ltd., shanghai, China) used as a dispersing agent). Both pure Cu and Cu-0.5 wt.% TiH_2_ green samples were produced by cold pressing (Ø10 mm) in a floating cylindrical die under 400 MPa. The green density was calculated by measuring dimensions and weight of the specimens. Cu samples were then heated in a dilatometer (DIL, Netzsche 402C, Freistaat Bayern, Germany) at a rate of 10 °C/min under flowing Ar to different temperatures (850, 900, 920, 930 and 950 °C), soaked at the related temperatures for 2 h, and cooled down to the room temperature at the same rate. The sintered samples were then polished and etched with a solution containing 5 g FeCl, 50 mL HCl and 100 mL H_2_O solution. The microstructures of both etched samples and as-polished samples were observed using an optical microscope (OM, Zeiss Axio Observer 3, Oberkochen, Germany). The OM micrographs were analyzed using image analysis software (ImageJ 1.52a, America) to reveal the grain size and porosity of the sintered samples. In order to investigate the effects of TiH_2_ on the sintering behavior of Cu, a spontaneous thermal analyzer (STA, Netzsche 449, Freistaat Bayern, Germany) was used to conduct Thermos-gravimetric (TG, Netzsche 449, Freistaat Bayern, Germany) and differential scanning calorimetric (DSC, Netzsche 449, Freistaat Bayern, Germany) analysis on the TiH_2_ powder, when it was heated from room temperature to 950 °C at a rate of 10 °C/min under flowing argon. Some of the Cu-0.5 wt.% TiH_2_ samples were deliberately isothermally treated at 650 °C for 0.5, 1 and 1.5 h respectively, before being heated to the sintering temperature to ensure enough time for TiH_2_ to react with copper oxide. The relative density was determined by the ratio of sintered density as measured to the theoretical density calculated by the rule of mixtures. Oxygen content of the as-sintered samples was measured by Oxygen/Nitrogen analyzer (Leco ON736, Saint Joseph, MI.USA). The microstructures were observed using a scanning electron microscope (SEM, Zeiss EVO-10, Oberkochen, Germany).

## 3. Experimental Results 

**Figure 1:**
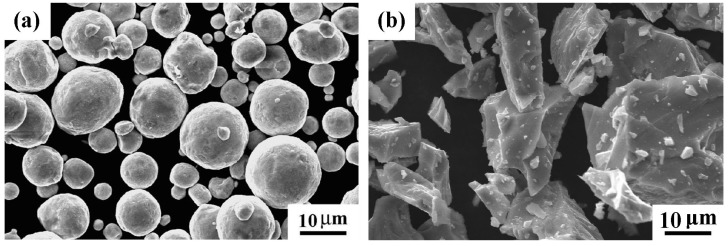
The microstructure of the powders: (**a**) Cu; and (**b**) TiH_2_.

The SEM micrographs of the Cu and TiH_2_ powders are shown in Figure 1. The Cu powder is in a spherical shape but the TiH_2_ powder has an irregular morphology. 

**Figure 2:**
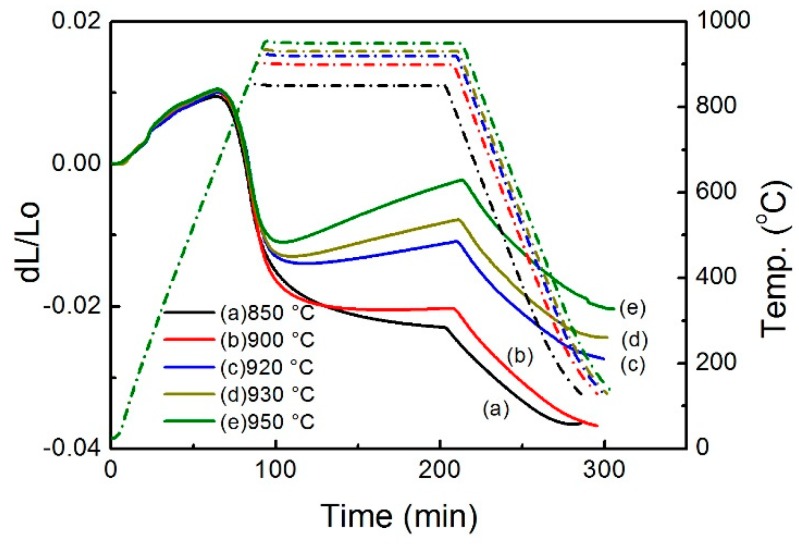
The dilatometry curves of Cu samples sintered at different temperatures.

Figure 2 shows the dilatometer (DIL) curves of green samples of Cu sintered at different temperatures (850, 900, 920, 930 and 950 °C) for 2 h. At low temperatures, the dimensions of all the samples increase with increasing temperature due to heat expansion. However, the expansion slows down at ~600 °C, and subsequently changes to shrinkage when the samples start to sinter. Cu samples sintered at different temperatures show quite different sintering behaviors. The sample sintered at 850 °C shrinks continuously during the sintering process, although the shrinkage rate gradually decreases with prolonging sintering time when the density of the sample is close to the theoretical density of Cu. As a comparison, the sample sintered at 900 °C has shorter shrinkage time. Its dimension remains almost unchanged in the late stage of soaking. Furthermore, the samples sintered at 920, 930 and 950 °C all exhibit expansion during high temperature soaking. Their expansion rates increase with increasing sintering temperature. The influences of sintering temperature on the sintering behaviors of Cu are reflected by the sintered densities of these samples, which are shown in Table 1. When the temperature increases from 850 to 950 °C, the sintered density decreases from 8.33 to 8.0 g/cm^3^. Accordingly, the relative density of the sintered samples decreases from 92.9% to 89.3%. As can be seen from Table 1, oxygen content increases continuously with increasing sintering temperature, and the sample sintered at 850 °C shows the minimum value of 1750 ppm oxygen content.

**Figure 3:**
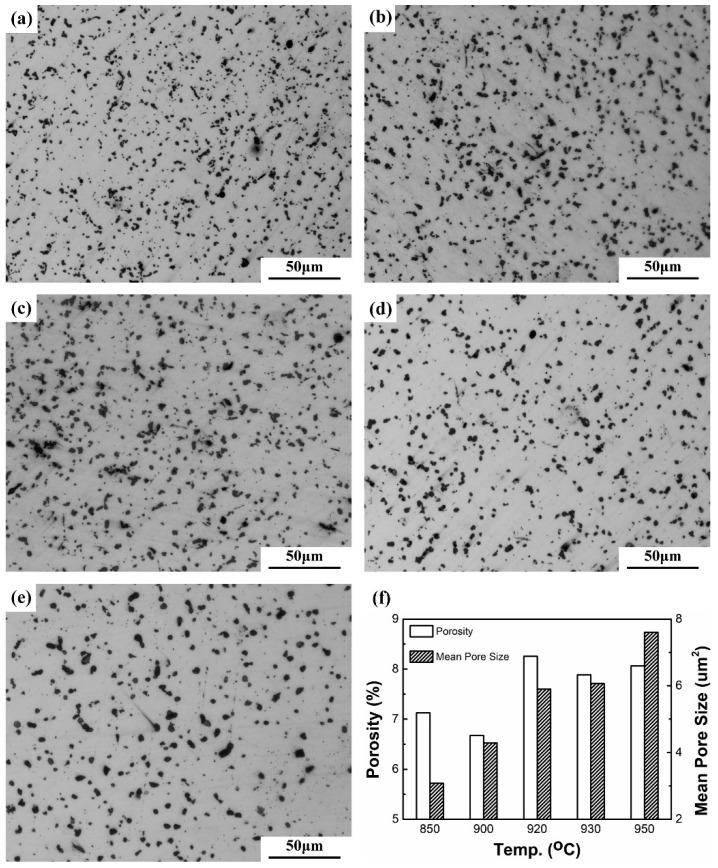
Optical micrographs of un-etched Cu samples sintered at (**a**) 850 °C, (**b**) 900 °C, (**c**) 920 °C, (**d**) 930 °C and (**e**) 950 °C. (**f**) The porosity content and mean pore sizes of the samples.

The optical micrographs of the samples sintered at different temperatures are shown in Figure 3, which reveals the pore structures at different samples. It can be seen that results accord well with the DIL curves and density measurements. Both the porosity and the average pore size of samples increased with increasing sintering temperature. The micrographs are analyzed using image analysis and the results are shown in Figure 3f. The average pore size of the sample sintered at 850 °C is only 3.1 μm, but it increases to 7.6 μm after sintering at 950 °C. 

**Figure 4:**
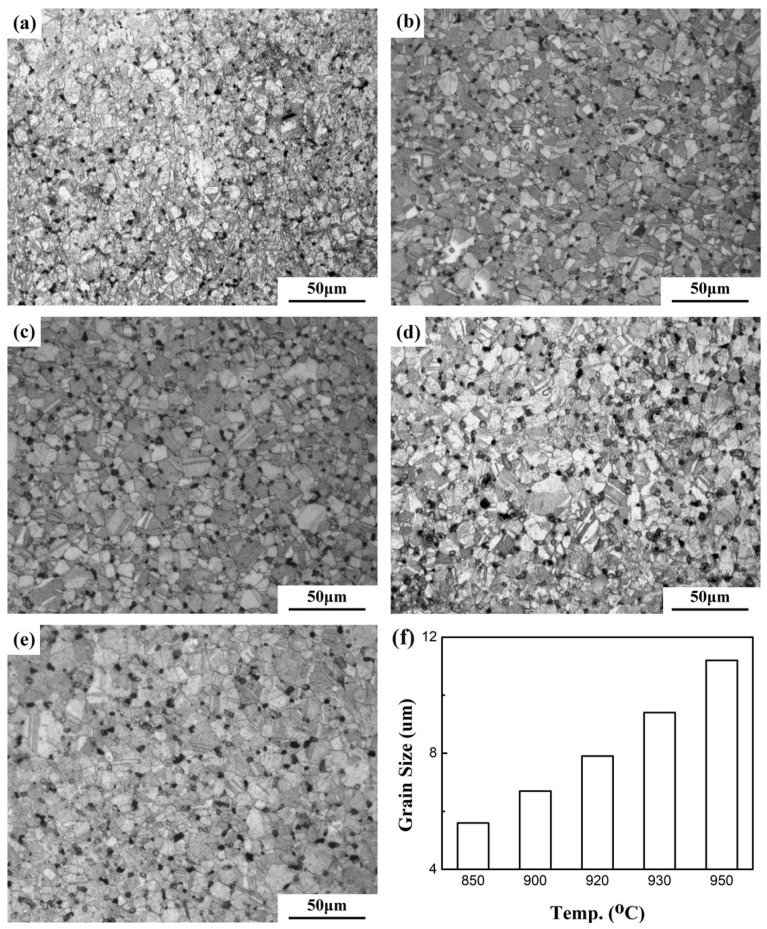
Optical micrographs of etched Cu samples sintered at (**a**) 850 °C, (**b**) 900 °C, (**c**) 920 °C, (**d**) 930 °C, (**e**) 950 °C, and (**f**) grain size of the samples versus sintering temperature.

In order to reveal grain structures, the sintered samples were etched and observed by an optical microscope. Figure 4 shows the microstructures of sintered samples at different temperatures after etching. It can be seen from Figure 4 that all the samples showed equiaxed grains and the grain size increased with increasing sintering temperature. The grain size of samples after sintering at different temperatures is plotted in Figure 4f. It can be seen from Figure 4f that the grain size of the sample sintered at 850 °C is only 5.6 μm, while the grain size of the sample sintered at 950 °C increases to 11.2 μm.

**Figure 5:**
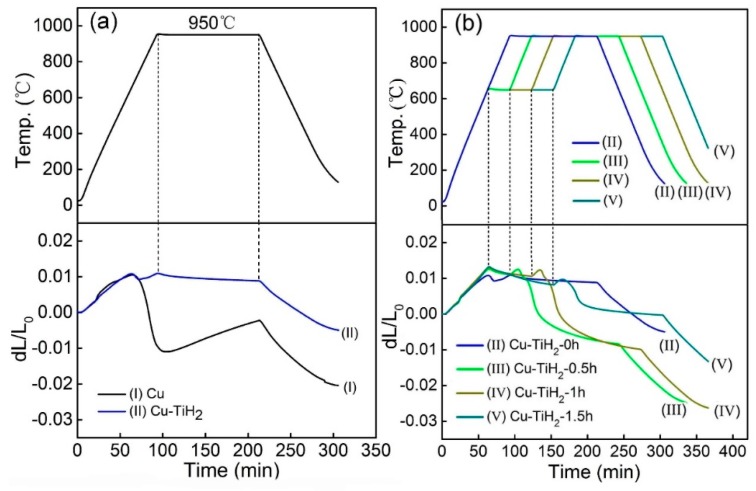
Dilatometry curves and corresponding temperature profiles of (**a**) pure Cu and Cu-0.5 wt.% TiH_2_ samples heated to 950 °C without isothermal treatment, and (**b**) Cu-0.5 wt.% TiH_2_ samples isothermally-treated at 650 °C for different time before being heated to 950 °C (dL/L_0_ is the ratio of the change in the length of the sample in the heating to its original length).

Figure 5 compares the dilatometry curves and corresponding temperature profile of the pure Cu and Cu-0.5 wt.%TiH_2_ samples. As it can be seen from Figure 5a, the shrinkage of pure copper sample happens soon after heating and then it changes to expansion however, the samples containing TiH_2_ show a different scenario. The expansion during the 950 °C isothermal treatment, which happens in the pure Cu sample, is absent from the Cu-0.5 wt.% TiH_2_ sample. However, the shrinkage which occurs in the early sintering stage of the pure Cu sample does not appear in the sintering of Cu-0.5 wt.% TiH_2_ sample either. Those differences imply different sintering mechanisms between the pure Cu and Cu-0.5 wt.% TiH_2_ samples. In order to clarify the influences of TiH_2_ on the sintering of Cu, the Cu-0.5 wt.% TiH_2_ samples held at 650 °C for 0.5, 1 and 1.5 h before heating to 950 °C. This isothermal treatment is designed to ensure a complete reaction between the hydrogen and the oxygen in the samples. The corresponding DIL curves are shown in Figure 5b. The DIL curves of the TiH_2_ containing samples are quite different from the TiH_2_ free samples, and shrinkage can be found in the isothermal treatment stage of the TiH_2_ containing samples. The samples exhibit two expansion peaks before the dilatometry curve finally changes to shrinkage. Table 2 lists the sintered and relative densities, oxygen content and temperatures corresponding to the first peak (TP_1_) and the second peak (TP_2_) for the Cu-0.5 wt.% TiH_2_ samples after sintering at different conditions. The temperature of the first peak (TP_1_) is ~650 °C, which varies little with isothermal treating condition. The temperature of the second peak (TP_2_) becomes lower after the sample is isothermally treated. It decreases with increasing isothermal treating time.

**Figure 6:**
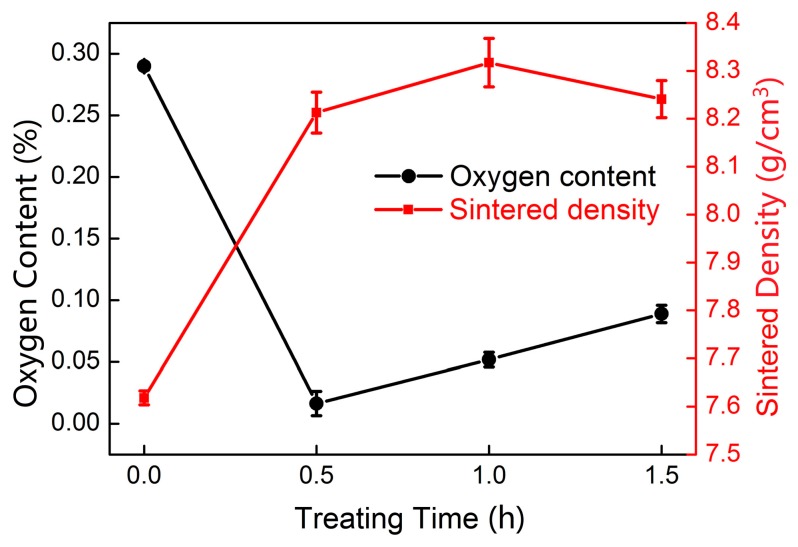
Oxygen contents and sintered density of Cu-0.5 wt.% TiH_2_ samples treated at 650 °C for different time.

The sintered density and oxygen contents of the sintered Cu-0.5 wt.% TiH_2_ samples were measured. The results are listed in Table 2 and shown in Figure 6. The density of Cu-0.5 wt.% TiH_2_ sintered at 950 °C without isothermal treatment is 7.62 g/cm^3^, which is lower than that of the pure Cu sample sintered at the same temperature (8.00 g/cm^3^). This suggests that the TiH_2_ has adverse effect on the sintering of Cu. However, the sintering behaviors of the Cu-0.5 wt.% TiH_2_ samples can be significantly improved by isothermal treatment at 650 °C. As it can be seen from Figure 6, the three isothermally treated samples all exhibit higher sintered densities than the sample which is not isothermally treated. Particularly, the sample treated at 650 °C for 1 h exhibits the highest density (8.31 g/cm^3^), which is higher than the pure Cu sample (8.00 g/cm^3^). This result indicates that doping with TiH_2_ can facilitate the sintering of Cu effectively if the TiH_2_-doped samples are properly treated. Apart from sintered density, the isothermal treatment influences the oxygen contents of the sintered samples as well. The Cu-0.5 wt.% TiH_2_ sample without isothermal treatment shows a very high amount of oxygen content (2900 ppm), which is even higher than the pure Cu sample sintered at the same temperature. However, the sample isothermally treated at 650 °C for 0.5 h has the oxygen content reduced to 160 ppm. Though the oxygen content of Cu-0.5 wt.% TiH_2_ samples isothermally treated at 650 °C increases with treating time. The sample treated for 1.5 h still has much lower oxygen content (890 ppm) than either the un-treated sample (2900 ppm) or the pure Cu sample (2130 ppm).

**Figure 7:**
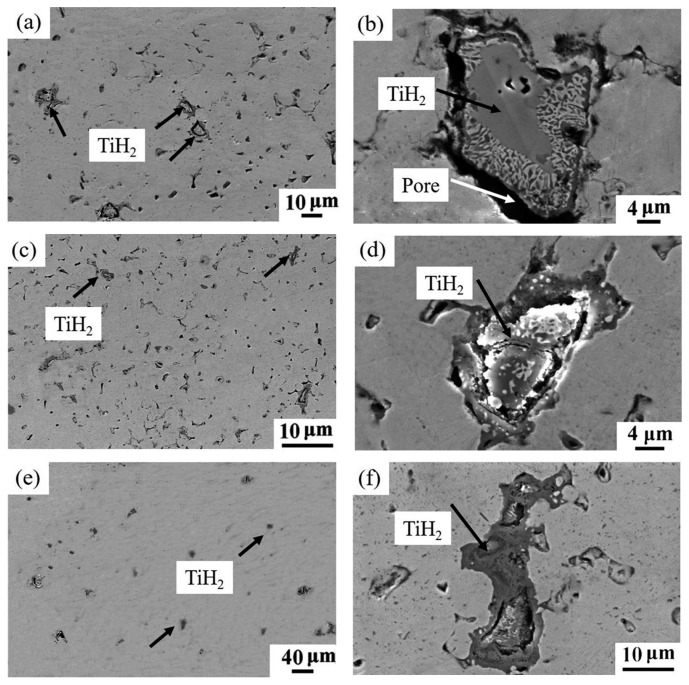
SEM micrographs of Cu-0.5 wt.% TiH_2_ samples sintered at 950 °C (**a**) without isothermal treatment; (**c**) isothermally treated at 650 °C for 0.5 h, (**e**) isothermally treated at 650 °C for 1 h, (**b**), (**d**) and (**f**) are higher magnification of the figures show in (a,c,e), respectively.

The microstructures of the sintered Cu-0.5 wt.% TiH_2_ samples without isothermal treatment and those treated at 650 °C for 0.5 and 1 h are shown in Figure 7. It is noted that big cracks exist along the Cu/TiH_2_ interfaces of the untreated samples (Figure 7b). However the size of these cracks becomes smaller in the sample isothermally treated for 0.5 h (Figure 7d) and completely disappears in the sample treated for 1 h (Figure 7f).

## 4. Discussion

**Figure 8:**
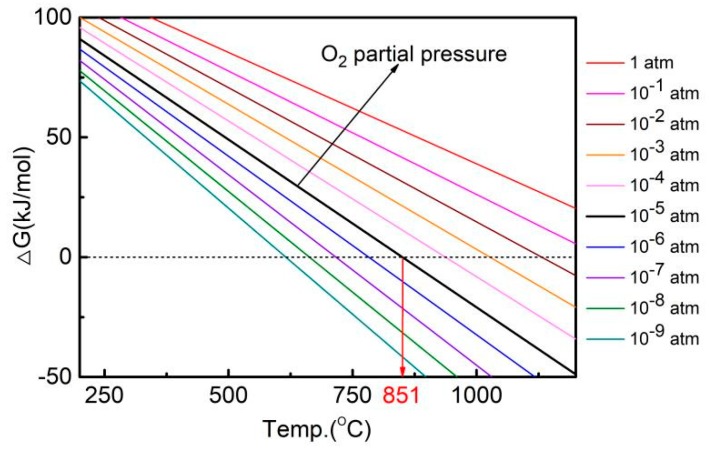
The Gibbs free energy of the reaction under different partial pressure of oxygen.

Cu powder used in the experiment is fabricated by water atomization. Some Cu get oxidized during the fabrication. Therefore, the Cu powder contains ~5000 ppm oxygen. The Cu oxide is not stable during sintering and tends to decompose during sintering through the following reaction [28]:

(1) $$CuO\left(s\right)=Cu\left(s\right)+\;\frac{1}{2}\;O_{2}\;\left(g\right)$$

where s and g stand for solid and gas states of the substances, respectively. Based on the enthalpy and entropy data listed in Table 3, the Gibbs free energy of the reaction under different partial pressure of oxygen can be calculated. Figure 8 shows a plot of the Gibbs free energy versus the temperature for the reaction at different partial pressures of oxygen. The Gibbs free energy of the reaction decreases with increasing temperature and becomes negative at elevated temperatures, which means that the CuO decomposes spontaneously when the temperature is high enough. The intersection between the Gibbs free energy curve and the horizontal broken line (where ∆G = 0) thus represents the temperature at which CuO starts to decompose. Apparently, this temperature increases with increasing oxygen partial pressure, implying that the CuO is more stable and only decomposes at higher temperatures when the oxygen partial pressure becomes higher. The argon used in the experiment contains ~10 ppm oxygen, which gives an oxygen partial pressure of ~10^−5^ atm during sintering. Under this condition, the CuO starts to decompose at ~851 °C, which produces oxygen in the sample. During sintering, the oxygen may be trapped in the closed pores and pressure in the pores builds up when the reaction proceeds. As a result, the sintering process is interrupted by the inflating pores, and the shrinkage slows down and finally turns to expansion. As seen in Figure 2, when the pure Cu samples are sintered at different temperatures, only the one sintered at 850 °C keeps shrinking throughout the isothermal sintering, while the others show different degrees of expansion. The higher the sintering temperature, the larger expansion is. 

This situation is changed with the involvement of TiH_2_. It is reported that when Cu is sintered in a hydrogen-containing atmosphere, the copper oxide can be reduced by hydrogen and water vapor is generated [21]. The reaction is as following [29]:

(2) $$CuO\left(s\right)+\;H_{2}\;\left(g\right)=Cu\left(s\right)+H_{2}O\;\left(g\right)$$

**Figure 9:**
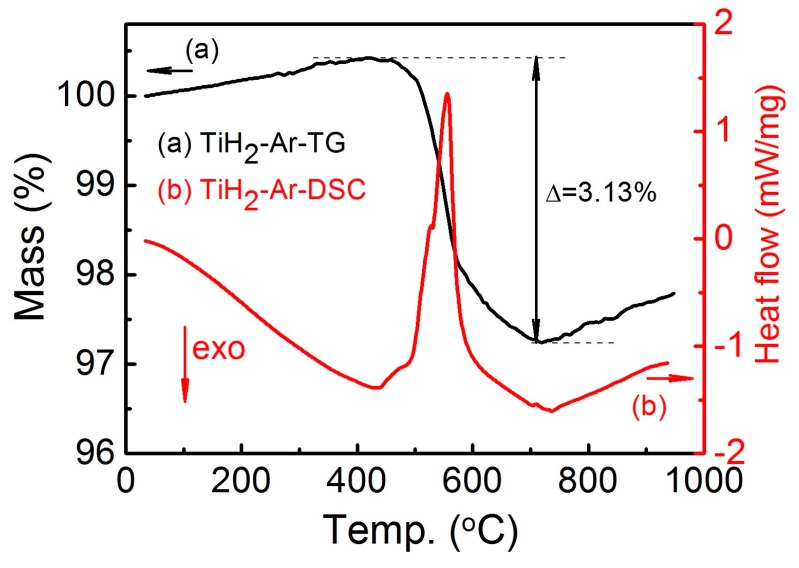
The (**a**) TG and (**b**) DSC of TiH_2_ powder heated to 1000 °C under flowing Ar.

During sintering of the Cu-0.5 wt.% TiH_2_ samples, the hydrogen of the above reaction can be provided by the decomposition of TiH_2_. Figure 9 shows the DSC and TG results of TiH_2_ powders heated from room temperature to 950 °C under flowing Ar. TiH_2_ exhibits a dramatic weight loss ~3.1% in the temperature range of 450~700 °C, which is in consistent with previous findings [30,31,32]. DSC analysis reveals the existence of endothermic peaks corresponding to the decomposition of TiH_2_ in a similar temperature range. The profile of mass loss was also reported in the work of Jiménez et al. [33], Matijasevic-Lux et al. [34] and Liu et al. [35]. They report that the mass loss corresponds to a series of dehydrogenation reactions in which the TiH_2_ is transformed to α-Ti phase.

When the DSC and TG results of TiH_2_ powders are compared with the dilatometry curves of Cu-0.5 wt.% TiH2 samples, it is found that the temperature of the first peak in the dilatometry curve of the Cu-0.5 wt.% TiH_2_ samples (TP_1_ ~650 °C) is within the temperature range of TiH_2_ decomposition. Therefore, it is reasonable to presume that the first shrinkage in the DIL curves is caused by the decomposition of TiH_2_, producing hydrogen and reducing the oxide in the Cu particles subsequently. This claim has been confirmed by the research conducted by Oanh et al. [36] which showed that oxygen in the in-situ formed Cu-TiC composite can be reduced by the addition of a small amount of TiH_2_.

At the early stage of sintering, most of the pores in the samples are open, therefore the hydrogen and water vapor can flow out of the material through the interconnected channels of the open pores. However, if the heating rate is so fast that the TiH_2_ cannot decompose completely before pores in the sintered material are closed up at higher temperatures, the hydrogen and water vapor will be trapped inside the closed pores, affecting the densification process of copper. As the temperature increases, the pressure of water vapor enveloped inside the closed pores builds up, inflating the pores and reversing the trend of shrinkage. Therefore, the sample starts to expand again and does not resume the shrinkage process until at an even higher temperature the sintering kinetic speeds up so much that the expansion caused by the inflating pores is offset by the sintering of Cu. As a result, two expansion peaks are found in the DIL curves of the Cu-0.5 wt.% TiH_2_ samples. The hydrogen trapped inside the closed pores not only results in low sintered density but also leads to an incomplete reduction of oxygen in the Cu, as evidenced by the high oxygen content of the untreated sample. However, this situation can be changed by an isothermal treatment at 650 °C. Because this temperature is higher than the decomposition temperature of TiH_2,_ but lower than the sintering temperature of Cu (775 °C based on the DIL curve of the pure Cu sample), the sample does not get significantly sintered and open pores in the sample do not close up in the isothermal treatment. Therefore, hydrogen can move quickly in the sample and reduce oxygen in the Cu effectively. Moreover, water vapor (the reaction product), can dissipate freely through the interconnected channels of the open pores from the sample. After the isothermal treatment, the oxygen content in the sample is already very low. Therefore, samples can be sintered to a high density when the temperature increases to 950 °C in the late stage.

## 5. Conclusions

In this research, we investigate the sintering behavior of Cu powder with 5000 ppm oxygen content and the influence of small amounts of TiH_2_ added to the sintering process.

(1) The oxide in the Cu powder decomposes when the sintering temperature increases to ~850 °C. The reaction product, oxygen, is trapped inside the closed pores during sintering. The inflation effect interrupts the densification of Cu, resulting in an expansion of the sample at temperatures above 850 °C. When the samples are sintered at temperatures between 850 and 950 °C, the sintered density decreases with sintering temperature, while the grain size increases with sintering temperature.

(2) The sintering behavior of Cu can be changed when 0.5 wt.% TiH_2_ is added into Cu. The TiH_2_ gradually decomposes from 450 to 700 °C. Hydrogen is produced in the reaction, which can reduce the oxide in the Cu powder at relatively low temperatures. However, if the Cu sample is sintered directly to 950 °C, Cu gets sintered quickly, the hydrogen and water vapor tend to be trapped in the closed pores, which results in an even lower sintering density than the pure Cu sample.

(3) This situation can be changed by isothermally treating the samples at 650 °C for 0.5, 1 and 1.5 h before heating them to 950 °C. The treating temperature is sufficient for oxide in the Cu to be reduced but not high enough for closed pores to form, so that the gas products can dissipate freely from the sample through the interconnected channels. Therefore, the isothermal treatment ensures a complete reaction between oxide and hydrogen. As a result, the isothermally treated Cu-0.5 wt.% TiH_2_ samples have lower oxygen contents and improved sintered densities compared to pure Cu sample.

## Figures and Tables

**Table 1:** Influence of sintering temperature on the density, porosity content, grain size and oxygen content of copper.

Sintering Temperature (°C)	Sintered Density, g/cm^3^	Relative Density, %	Porosity, %	Mean Pore Size, μm	Grain Size, μm	Oxygen Content, ppm
**850**	8.33	92.9	7.12	3.1	5.6	1750
**900**	8.40	93.8	6.68	4.3	6.7	2020
**920**	8.01	89.7	8.25	5.9	7.9	2580
**930**	8.10	90.4	7.88	6.1	9.4	2240
**950**	8.00	89.3	8.06	7.6	11.2	2130

**Table 2:** A summary of densities, oxygen contents and expansion peak temperatures of the sintered samples of Cu-0.5 wt. %TiH_2_ hold at 650 °C for different time.

Samples	Sintered Density, g/cm^3^	Relative Density, %	Oxygen Content, ppm	Tp_1_, °C	Tp_2_, °C
**Cu-TiH**_**2**_ **-0 h**	7.62	85.0	2900	649	952
**Cu-TiH**_**2**_ **-0.5 h**	8.21	91.8	160	650	762
**Cu-TiH**_**2**_ **-1 h**	8.32	93.1	520	650	770
**Cu-TiH**_**2**_ **-1.5 h**	8.24	90.9	890	650	775

**Table 3:** Free energy, enthalpy and entropy of the reaction CuO = Cu + 1/2 O_2_ at different partial pressure of oxygen.

Pressure/atm	∆H (kJ/mol)	∆S (kJ/mol K)	∆G = ∆H−T∆S (kJ/mol)
1	157.3	0.093	∆G = 157.3 − 0.093 T
10^−1^		0.103	∆G = 157.3 − 0. 103 T
10^−2^		0.112	∆G = 157.3 − 0.112 T
10^−3^		0.121	∆G = 157.3 − 0.121 T
10^−4^		0.130	∆G = 157.3 − 0.130 T
10^−5^		0.140	∆G = 157.3 − 0. 140 T
10^−6^		0.149	∆G = 157.3 − 0.149 T
10^−7^		0.159	∆G = 157.3 − 0.159 T
10^−8^		0.168	∆G = 157.3 − 0.168 T
10^−9^		0.177	∆G = 157.3 − 0.177 T

## References

[1] Krishnan S., Haseeb A.S.M.A., Johan M.R. (2012). Preparation and Low-Temperature Sintering of Cu Nanoparticles for High-Power Devices. IEEE Trans. Componen. Packaging Manuf. Technol..

[2] Sundaram R., Yamada T., Hata K., Sekiguchi A. (2017). Electrical performance of lightweight CNT-Cu composite wires impacted by surface and internal Cu spatial distribution. Sci. Rep..

[3] Ren S.B., Chen J.H., He X.B., Qu X.H. (2018). Effect of matrix-alloying-element chromium on the microstructure and properties of graphite flakes/copper composites fabricated by hot pressing sintering. Carbon..

[4] Han L.T., Liu J.W., Tang H.G., Ma X.F., Zhao W. (2018). Investigation on the properties of nanostructured Cu alloy prepared by mechanical milling and reactive hot-pressing. J. Alloy Compd..

[5] Yao G.C., Mei Q.S., Li J.Y., Li C.L., Ma Y., Chen F., Liu M. (2016). Cu/C composites with a good combination of hardness and electrical conductivity fabricated from Cu and graphite by accumulative roll-bonding. Mater. Des..

[6] Zhao S., Zheng Z., Huang Z.X., Dong S.J., Luo P., Zhang Z., Wang Y.W. (2016). Cu matrix composites reinforced with aligned carbon nanotubes: mechanical, electrical and thermal properties. Mater. Sci. Eng. A.

[7] Shaik M.A., Golla B.R. (2019). Development of highly wear resistant Cu-Al alloys processed via powder metallurgy. Tribol. Int..

[8] Souli I., Gruber G.C., Terziyska V.L., Zechner J., Mitterer C. (2018). Thermal stability of immiscible sputter-deposited Cu-Mo thin films. J. Alloy Compd..

[9] Vincent C., Silvain J.F., Heintz J.M., Chandra N. (2012). Effect of porosity on the thermal conductivity of copper processed by powder metallurgy. J. Phys. Chem. Solids..

[10] Schubert Th., Trindade B., Weißgärber T., Kieback B. (2008). Interfacial design of Cu-based composites prepared by powder metallurgy for heat sink applications. Mater. Sci. Eng. A..

[11] Upadhyaya A., Upadhyaya G.S. (1995). Sintering of copper-alumina composites through blending and mechanical alloying powder metallurgy routes. Mater. Des..

[12] Deng Z.H., Yin H.Q., Zhang C., Zhang G.F., Li W.Q., Zhang T., Zhang R., Jiang X., Qu X. (2019). Microstructure and mechanical properties of Cu–12Al–xNi alloy prepared using powder metallurgy. Mater. Sci. Eng. A..

[13] Paderin S.N., Shil’nikov E.V. (2015). Thermodynamic laws of the oxygen solubility in liquid metals (Ni, Co, Fe, Mn, Cr) and the formation of oxygen-containing solutions in the alloys based on them. Russian Metall..

[14] Tang R.Z., Tian R.Z. (2009). Binary Alloy Phase Diagrams and Crystal Structure of Intermediate Phase.

[15] Guo R.Q., Rohatgi P.K., Nath D. (1997). Preparation of aluminium-fly ash particulate composite by powder metallurgy technique. J Mater. Sci..

[16] Yu P., Qian M., Li L., Schaffer G.B. (2010). On the infiltration mode during fabrication of aluminium composite. Acta Mater..

[17] Homeny J., Buckley M.M. (1991). Transmission electron microscopy study of an aluminum oxide fiber/aluminum-magnesium alloy metal matrix composite interface. Mater. Lett..

[18] Lumley R.N., Sercombe T.B., Schaffer G.B. (1999). Surface oxide and the role of magnesium during the sintering of aluminum. Metall. Mater. Trans. A..

[19] Mcleod A.D., Gabryel C.M. (1992). Kinetics of the growth of spinel, MgAl2O4, on alumina particulate in aluminum alloys containing magnesium. Met. Trans. A..

[20] Mahmoud M.M., Link G., Thumm M. (2015). The role of the native oxide shell on the microwave sintering of copper metal powder compacts. J. Alloy Compd..

[21] Ramakrishnan P., Tendolkar G.S. (1964). Influence of thin oxide films on the mechanical properties of sintered metal-powder compacts. Powder Metall..

[22] Li W.J., Shao W.Z., Xie N., Zhang L., Li Y.R., Yang M.S., Chen B.A., Zhang Q., Wang Q., Zhen L. (2018). Air arc erosion behavior of CuZr/Zn2SnO4 electrical contact materials. J. Alloy Compd..

[23] Zhao L., Zhang X.H., Deng T.Q., Jiang J. (2018). Develop an effective oxygen removal method for copper powder. Adv. Powder Technol..

[24] Hao H.L., Mo W., Lv Y.H., Ye S.L., Gu R.N., Yu P. (2016). The effect of trace amount of Ti and W on the powder metallurgy process of Cu. J. Alloy Compd..

[25] Hao H.L., Ye S.L., Yu K.P., Chen P., Gu R.N., Yu P. (2016). The role of alloying elements on the sintering of Cu. J. Alloy Compd..

[26] Wang C.M., Pan L., Zhang Y., Xiao S., Chen Y. (2016). Deoxidization mechanism of hydrogen in TiH_2_ dehydrogenation process. Int. J. Hydrogen Energy..

[27] Leipunsky I.O., Zhigach A.N., Kuskov M.L., Berezkina N.G., Afanasenkova E.S., Kudrov B.V., Lopez G.W., Vorobjeva G.A., Naumkin A.V. (2019). Synthesis of TiH_2_ nano powder via the guen-miller flow-levitation method and characterization. J. Alloys Comp..

[28] Lide D.R., Lide K.S. (2010). The Handbook of Chemistry and Physics.

[29] Rodriguez J.A., Kim J.Y., Hanson J.C., Pérez M., Frenkel A.I. (2003). Reduction of CuO in H_2_: In Situ Time-Resolved XRD Studies. Catal. Letters..

[30] Bhosle V., Baburaj E.G., Miranova M., Salama K. (2003). Dehydrogenation of TiH_2_. Mater. Sci. Eng. A..

[31] German R. (2014). Sintering: from Empirical Observations to Scientific Principles.

[32] Azevedo C.R.F., Rodrigues D., Neto F.B. (2003). Ti–Al–V powder metallurgy (PM) via the hydrogenation–dehydrogenation (HDH) process. J. Alloys Comp..

[33] Jiménez C., Garcia-Moreno F., Rack A., Tucoulou R., Klaus M., Pfretzschner B., Rack T., Cloetens P., Banhart J. (2012). Partial decomposition of TiH_2_ studied in situ by energy-dispersive diffraction and ex situ by diffraction microtomography of hard X-ray synchrotron radiation. Scr. Mater..

[34] Matijasevic-Lux B., Banhart J., Fiechter S., Görke O., Wanderka N. (2006). Modification of titanium hydride for improved aluminium foam manufacture. Acta Mater..

[35] Liu H., He P., Feng J.C., Cao J. (2009). Kinetic study on nonisothermal dehydrogenation of TiH_2_ powders. Int. J. Hydrogen Energy..

[36] Oanh N.T.H., Viet N.H., Kim J.S., Junior A.M.J. (2017). Characterization of In-Situ Cu–TiH2–C and Cu–Ti–C Nanocomposites Produced by Mechanical Milling and Spark Plasma Sintering. Metals..

